# Effects of an additional multimodal intervention (MultiMove) during inpatient rehabilitation on clinical and functional outcomes in patients with chronic low back pain– a pilot trial

**DOI:** 10.1186/s12891-025-08494-2

**Published:** 2025-04-16

**Authors:** Toan Nguyen, Martin Behrens, Kim-Charline Broscheid, Robert Bielitzki, Kerstin Rohkohl, Ivonne Rudolph, Katharina Meiler, Jörg Franke, Lutz Schega

**Affiliations:** 1 Department of Sport Science, Institute III, Otto von Guericke University Magdeburg, Magdeburg, Germany; 2 University of Applied Sciences for Sport and Management Potsdam, Olympischer Weg 7, 14471 Potsdam, Germany; 3 Department of Orthopaedics, Rostock University Medical Centre, Rostock, Germany; 4 Department of Sport and Exercise Medicine, Institute of Movement Science and Psychology, University of Hamburg, Hamburg, Germany; 5 Rehabilitation Clinic Bad Salzelmen, Waldburg Zeil Clinics, Schönebeck, Germany; 6 Department of Orthopedic Surgery, Klinikum Magdeburg gGmbH, Magdeburg, Germany

**Keywords:** Dancing, Functional mobility, Physical functions, Cognitive functions, Quality of life, Walking, Kinesiophobia, Depression

## Abstract

**Background:**

As a leading cause of disability, chronic low back pain (CLBP) is a musculoskeletal condition often associated with impaired physical and cognitive functions. Due to its multi-factorial facets, the application of multimodal interventions is recommended. MultiMove is a multimodal intervention designed for CLBP patients, which combines motor-cognitive and dancing exercises. This study aimed to assess the effects of an additional MultiMove intervention to a standard inpatient rehabilitation on clinical and functional outcomes in CLBP patients.

**Methods:**

For this prospective, two-arm, controlled pilot trial, 27 CLBP patients (17 females, 10 males) undergoing a 3-week inpatient rehabilitation, in a rehabilitation clinic in Germany, were recruited. The intervention group (IG, *n* = 15, 61.6 ± 1.8 years) received a daily MultiMove session in addition to the standard rehabilitation, while the control group (CG, *n* = 12, 63.8 ± 2.2 years) followed the standard rehabilitation. Physical (Timed Up and Go (TUG) [primary outcome], Five-Repetition Sit-to-Stand (FRSTS), and Six-Minute Walk Test (6MWT), trunk range of motion, single and dual task walking)), clinical (acute/chronic pain intensity, Oswestry Disability Index, EQ-5D-5 L), cognitive (Stroop Color Word Test, Trail Making Test), and psychosocial outcomes (Tampa Scale of Kinesiophobia, Beck Depression Inventory-II, Coping Strategies Questionnaire) were assessed during the first (pre-test) and last day (post-test) of the inpatient rehabilitation.

**Results:**

The statistical analyses revealed improvements in trunk range of motion (sagittal plane: *p* = 0.018, *d* = 1.00; transversal plane: *p* = 0.006, *d* = 1.18) and 6MWT performance (*p* = 0.003, *d* = 1.30) in the IG compared to the CG. Moreover, lower dual task costs for a gait variability measure (*p* = 0.034, *d* = 0.97) as well as reduced chronic pain intensity (*p* = 0.004, *d* = 1.33), kinesiophobia (*p* = 0.035, *d* = 1.15), and depression (*p* = 0.034, *d* = 1.08) were found in favour of the IG.

**Conclusion:**

Data indicate that the multimodal intervention MultiMove improved clinical and functional outcomes in CLBP patients during inpatient rehabilitation. Therefore, conducting a randomized controlled trial with a large sample size is recommended to verify and extent these results.

**Trial registration:**

MultiMove project (German Clinical Trial Register, ID: DRKS00021696 / 10.07.2020, https://drks.de/drks_web/navigate.do?navigationId=trial.HTML26TRIAL_ID=DRKS00021696) and was carried out in the rehabilitation clinic Bad Salzelmen (Schönebeck, Germany).

## Introduction

Low back pain (LBP) is one of the highest cause of disability worldwide [1, 2] and affected 619 million people globally in 2020 [3]. While the majority of patients recover in the following weeks after the emergence of symptoms, LBP can persist in some cases. If the LBP lasts more than 12 weeks it is referred to as chronic low back pain (CLBP) [4]. This condition is often associated with an impaired motor control that can affect spine stability, postural control, flexibility [5, 6], and, consequently, deteriorates gait performance [7]. More precisely, there are several spatio-temporal gait parameters, such as stride length, gait velocity, minimum toe clearance and their respective variability, which were found to be impaired in CLBP patients and might increase the risk of falling [8, 9]. Moreover, CLBP has been shown to impair cognitive functions indicated by a poorer performance during executive function tests compared to healthy controls [10]. Besides, psychosocial factors, such as depression and kinesiophobia, were found to be associated with the development of pain chronicity [4]. Consequently, CLBP negatively impacts activities of daily living and thus quality of life [11].

Motor control and stabilization training is recommended for the rehabilitation of CLBP as these interventions have been shown to be most effective in reducing CLBP intensity [12]. However, the prescription of an appropriate intervention is often challenging [7]. Therefore, multimodal approaches were developed with promising results [13]. In this regard, it was recently suggested to combine motor-cognitive exercises, dance exercises, as well as conventional strength and flexibility training (termed MultiMove) to address the various impairments of CLBP patients [14]. The motor-cognitive exercises require simultaneous execution of motor, cognitive, and visual tasks with incremental difficulty, which can be beneficial for balance, stabilization, gait, and cognitive performance [14, 15]. Besides, dancing has not only been shown to induce favorable neurocognitive adaptations, such as improved motor control, proprioceptive skills, coordination, and cognition [16], but also to improve psychological and social health especially in group settings [17].

The MultiMove intervention was initially developed as an outpatient progressive long-term rehabilitation program (i.e., 12 weeks, 2 × 60 min per week). For this pilot study, the MultiMove intervention was adapted to be used in the early post-operative inpatient rehabilitation for patients with specific CLBP. Thus, the aim of this pilot study was to investigate the effects of a daily MultiMove intervention, in addition to the standard inpatient therapy executed in Germany, on functional mobility (primary outcome) as well as on pain intensity, trunk range of motion (ROM), leg extensor muscle power, exercise capacity, single and dual task walking performance, executive functioning, pain coping skills, psychosocial aspects, and quality of life (secondary outcomes). The standard inpatient therapy consisted of daily interventions, e.g., physical therapy, group therapy, medical training therapy, aqua fitness, and trunk muscle strengthening, performed for 3 weeks.

We hypothesized that the additional MultiMove intervention improves functional mobility, pain intensity, physical functions, gait and cognitive performance, as well as psychosocial aspects (e.g., kinesiophobia), disability, and quality of life to a greater extent than the standard inpatient therapy alone.

## Methods

### Participants and study design

This prospective, two-arm, controlled pilot study is part of the MultiMove project (German Clinical Trial Register, ID: DRKS00021696 / 10.07.2020, https://drks.de/drks_web/navigate.do?navigationId=trial.HTML26TRIAL_ID=DRKS00021696) and was carried out in the rehabilitation clinic Bad Salzelmen (Schönebeck, Germany).

Due to the pilot character of this study, no sample size calculation was conducted. Patients were included, when they complied with the following criteria: (i) ≥ 50 years old, (ii) LBP symptoms for more than 12 weeks, (iii) diagnosed to suffer from CLBP according to the International Classification of Diseases 10th revision (ICD-10: M54 Dorsalgia; M48.0 Spinal stenosis; M54.5 Low back pain; M54.4 Lumbago with sciatica; M54.1 Radiculopathy; M41.5 Other secondary scoliosis; M43.1 Spondylolisthesis; M42.1 Adult osteochondrosis of spine; M51.2 Other specified intervertebral disc displacement; M47.8 Other spondylosis; M53.2 Spinal instabilities). There was no inclusion criterion regarding pain intensity, as all patients were treated because of specific CLB in the inpatient rehabilitation clinic. Exclusion criteria were as follows: (i) dependence on a walking aid or inability to walk more than 300 m at a stretch, and (ii) any neurological, cardiovascular, psychological, and musculoskeletal diseases that preclude the execution of the intervention and the measurements.

### Study procedure

From May to June 2022, all CLBP patients who registered for an inpatient rehabilitation in the rehabilitation clinic Bad Salzelmen in Schönebeck, Germany were invited to take part in the study. On their admission day, eligible patients were assigned either to the intervention group (IG) or the control group (CG). Both groups followed the standard rehabilitation program of 3 weeks provided by the rehabilitation clinic. The IG additionally performed a MultiMove intervention on a daily basis for 30 min (Table 1). No randomization could be performed due to (i) the impossibility to foresee the exact number of incoming patients, (ii) the requirement of the MultiMove intervention to be performed as a group therapy, and (iii) the restricted schedule imposed by all active contributors (i.e., investigators, medical staff, physical therapists). Therefore, the patients were assigned to a group according to their admission day during the 7-week recruitment process (IG: week 1 to 4 and CG: week 5 to 7).

**Table 1:** Detailed description of the inpatient rehabilitation and MultiMove intervention including exercise examples.

	**Standard inpatient rehabilitation**									
	**Training**		**Volume**			**Setting**		**Description**		
	Spine mobilization		30 min, 2–3 x per week			Group therapy		Functional training • Mobilization, stabilization with small accessories (e.g., ball, foam cushion, etc.) • Exercises in all position (i.e., standing, sitting, lying)		
	Specific trunk muscle training		30 min, 2–3 x per week			One-to-one		Training focused on a specific position or functional activity • Sitting, lying, to carry an object, etc. Education • 2–3 weekly lectures of 45 min for e.g., back health, pain coping strategies for daily living, nutrition		
	Medical training therapy		60 min, 2–3 x per week			Individual		Personalized training program • Endurance and strength using fitness machines (e.g., cycle ergometer at low intensity (25 W) with 55–70 revolutions per min, treadmill at comfortable speed or fast walking, etc.)		
	Aqua fitness		30 min, 2–3 x per week			Group therapy		Coordination, stabilization, mobilization, strengthening, proprioception involving all major muscle groups		
	Physical therapy		30 min, 2 x per week			One-to-one		Thermotherapy, electrotherapy, massage (e.g., lymphatic drainage), taping mainly focusing on muscles of the lower extremities		
	Endurance training		60 min, 2–3 x per week			Group therapy		Walking or Nordic walking		
**MultiMove**										
				**Week 1** **Foundational**					**Week 2** **Intermediate**	**Week 3** **Advanced**
Motor-cognitive exercises*										
	Motor and cognitive tasks performed simultaneously with a high level of difficulty			Lean or step in different directions according to a wide variety of commands					Jumping Jack split into single parts (movement of only one limb at a time)	Independent movement or handling of at least two different limbs or objects, respectively, while reacting to external stimuli
Dancing										
	Different dance choreographies performed in a block formation			Line-Dance					Mambo	Line-Dance + Mambo

*Due to the trial-and-error character of the motor-cognitive exercise, no exact exercise parameters can be provided. After they were successfully completed, the exercises were made more difficult or changed.

On their day of arrival at the clinic, all patients were screened for eligibility by the medical staff. If the patients met the inclusion criteria and agreed to participate in the study, they were asked to fill a consent form. Afterwards, they were invited to take part in the pre-test. During the testing session, patients’ characteristics were recorded (e.g., age, height, weight, sex) and they had to perform two cognitive tests (i.e., Stroop Color and Word Test and the Trail Making Test) to assess executive functioning. Thereafter, the patients were equipped with two inertial measurement units (IMU), which were fixed to the top of each foot. They were then asked to complete three physical performance tests: (i) the trunk flexibility test, (ii) the Timed Up and Go (TUG) Test, and (iii) the Five-Repetition Sit-to-Stand (FRSTS) Test. Afterwards, the patients performed the following single and dual tasks in a randomized order: (i) single motor task (i.e., walking), (ii) single cognitive task (i.e., arithmetic task), and (iii) motor-cognitive dual task (walking + arithmetic task) to assess gait performance and dual task costs (DTC). The testing session terminated with the Six-Minute Walk Test (6MWT). At the end of the session, all patients received six questionnaires: (i) the German Pain Questionnaire, (ii) the Tampa Scale of Kinesiophobia, (iii) the Beck Depression Inventory-II, (iv) the Coping Strategies Questionnaire, (v) the Oswestry Disability Index, and (vi) the EuroQol Group’s EQ-5D-5 L to quantify the acute and chronic pain intensity, fear of movement in the context of pain, depression, effectiveness of pain coping strategies, deficits in physical and social functioning related to back pain, and quality of life, respectively. They were asked to fill them later during the day. All patients then followed their inpatient rehabilitation program according to the group assignment for 3 consecutive weeks. On their last day of rehabilitation, patients were invited to the post-test.

### Interventions

Prior to the start of the study, three volunteer therapists of the rehabilitation clinic were familiarized with the MultiMove intervention in four teaching units of 90–120 min each. During the 3-week inpatient rehabilitation phase, which represents the standard care in Germany, the participants of the IG participated in the MultiMove intervention 5 days per week for 30 min (30 min x 5 days x 3 weeks = 450 min). The intervention sessions started with motor-cognitive exercises followed by dance choreographies, each performed for 15 min with an incremental difficulty over time. Each training session was supervised by one instructor. All patients were initially asked to ensure an attendance rate of at least 80% (12 sessions). However, due to the German holidays (i.e., Mai 26 and June 6), the rate has been lowered to 60% (9 sessions) (9 × 30 min = 270 min). A detailed description of the inpatient rehabilitation and MultiMove intervention including exercise examples is provided in Table 1.

### Primary outcome

#### Functional mobility

Functional mobility was assessed with the TUG Test [18]. The patients started in a sitting position on a standardized chair (height: 44 cm) with the back and arms leaned on the backrest and armrests, respectively. At an acoustic start signal, the patients had to stand up without the help of their arms, walk straight 3 m, turn, walk back to the chair, and finally sit on the chair again without the help of their arms as fast as possible, while maintaining a safe pace. The test was repeated twice, and the mean time of both trials was used for data analysis.

### Secondary outcomes

#### Pain intensity

The German Pain Questionnaire was used for systematic assessment of the patient’s individual pain situation. In this regard, item 11 quantifies the acute and chronic pain intensity (i.e., the LBP intensity felt at the beginning of the testing session and the average LBP during the 4 previous weeks) using a numerical rating scale from 0 to 10 [19], which was included in the statistical analysis.

#### Trunk range of motion

The trunk flexibility test measured the trunk ROM using the mobee® med (SportMed A.G. SA, Luxembourg) in the sagittal, frontal, and transversal plane [20]. The measurements of the sagittal and frontal trunk ROM were performed in a standing position with both legs fully extended, while the transversal trunk ROM was quantified in a sitting position with the arms crossed in front of the chest. In addition, perceived pain intensity during each movement was also recorded using a numerical rating scale ranging from 0 to 10.

#### Leg extensor muscle power

The FRSTS Test is a functional performance test that measures the time to stand and sit five consecutive times. The patients were asked to sit on a standardized chair (chair height: 44 cm) with their arms crossed in front of the chest. At an acoustic signal, the participants completed the test as fast as possible. Care was taken that the feet stayed flat on the floor and the hips were fully extended during each repetition. The test was repeated twice, and the mean time of both trials was used for further analysis. Additionally, the relative sit-to-stand mean power was also calculated using the method suggested by Alcazar et al. [21].

#### Exercise capacity

The 6MWT is a safe and simple test used for the evaluation of exercise capacity [22]. During this test, the subjects are asked to reach the maximal walking distance within 6 min. In the present study, a straight track of 15 m marked every 1 m with a piece of tape has been set up and the patients were asked to walk back and forth as fast and safe as possible. The total distance was measured and included in the final analysis.

#### Single and dual task gait performance

Two 60-s trials were performed for each task and repeated twice. In the single motor task condition, the patients walked at their preferred gait velocity back and forth on a 15-m track. The spatio-temporal gait parameters were quantified with two IMUs (Xsens Technologies B.V., Netherlands). The IMUs were fixed on top of both feet with adhesive tape and the spatio-temporal gait parameters (i.e., stride length, gait velocity, minimum toe clearance, and their respective coefficient of variation (CoV)) were calculated according to the algorithm of Hamacher et al. [23]. All data were processed with MATLAB (MathWorks®, Version R2020b, Natick, USA). In the single cognitive task condition, a random number between 300 and 400 was given to the patients and they were asked to continuously subtract the value by three as fast as possible, while minimizing the number of mistakes. The final score was defined as the summation of all subtractions correctly performed during both trials. The motor-cognitive dual task involved the simultaneous execution of both the motor and cognitive tasks. The motor and cognitive DTC were calculated as follows:

$$\:\begin{array}{c}DTC=\frac{ST-DT}{ST}\times\:100\left(1\right)\end{array}$$

$$\:\begin{array}{c}DTC=\frac{DT-ST}{DT}\times\:100\left(2\right)\end{array}$$

Where $\:ST$ and $\:DT$ are the single and dual task performance respectively. The equation $\:\left(1\right)$ was used when a higher value reflected a better performance. Otherwise, the equation $\:\left(2\right)$ was used.

#### Executive functioning

The Stroop Color and Word Test was used to assess inhibitory control. At first, an example of all three conditions (i.e., (i) color words printed in black ink, (ii) the sign “XXXXX” printed in different ink colors, and (iii) color words printed in unmatched ink colors) was presented to the patients. Each example was composed of a sample of 15 items. For each condition, an A4 sheet containing five rows of 20 items was presented. The patients had 45 s to read or name all the presented words or colors, respectively. If every table was completed before the end of the given time, an estimated result was calculated as follows:

$$\:\begin{array}{c}estimated\:result=\frac{number\:of\:correct\:answers}{time\:required}\times\:45\left(3\right)\end{array}$$

The interference score was determined following the Golden’s method [24]. The test was repeated three times and an average score was calculated, which was used for data analysis.

The Trail Making Test Part A and B were used to assess cognitive flexibility. For the Part A, the numbers from 1 to 25 were randomly represented on an A4 sheet. The patients were asked to connect all numbers in ascending order with a pen and without lifting it. In Part B, the numbers 1 to 13 and letters A to L were randomly represented on an A4 sheet. Here, the patients were asked to alternatively connect all numbers in ascending order and letters in alphabetical order (i.e., 1-A-2-B-3-C-…). The patients were asked to first complete an example of each part (Part A: numbers 1 to 8; Part B: numbers 1 to 4 and letters A to D). The time required to complete each part as well as the time difference between the Part A and B was used for further analysis [25].

#### Pain coping skills, psychosocial aspects, disability, and quality of life

The Coping Strategies Questionnaire consists of 50 items and was used to evaluate six potential pain coping strategies (“diverting attention”, “reinterpreting pain sensations”, “coping self-statements”, “ignoring pain sensations”, “praying or hopping”, and “catastrophizing”), two behavioral coping strategies (“increasing activity level” and “increasing pain behavior”), and the effectiveness rating (“control over pain” and “ability to decrease pain”). All ratings were quantified on a scale from 0 to 6 [26]. The Tampa Scale of Kinesiophobia was used to measure the fear of movement via 11 items and the final score was calculated ranging from 11 to 44 [27]. The Beck Depression Inventory-II is a 21-items questionnaire that was used to assess the level of depression with scores ranging from 0 to 63 [28]. The Oswestry Disability Index is a 10-items questionnaire, which was used to quantify the level of disability in percent [29]. Lastly, the EQ-5D-5L was used to measure five aspects of the quality of life (“mobility”, “selfcare”, “activity”, “pain”, and “anxiety”) on a scale from 0 to 5, which are used to calculate a quality of life index [30]. Additionally, it also assesses the subjective health status through a visual analogue scale ranging from 0 to 100. All questionnaires have been shown to be valid and reliable [26, 27, 29–31].

### Statistical analysis

Only the data of patients who completed the pre- and post-test (IG: *n* = 15; CG: *n* = 12) were used for the statistical analysis. Normal distribution of data has been tested using the Shapiro-Wilk test. Given that no distribution have been found to be extremely skewed, analyses of covariance (ANCOVA) with baseline-adjustment have been conducted to compare the data of groups recorded at the post-test [32]. Differences were considered significant when *p* ≤ 0.05 (*: 0.01 < *p* ≤ 0.05; **: 0.001 < *p* ≤ 0.01; ***: *p* ≤ 0.001). Data are presented as means ± standard deviations (SD) and mean differences with 95% confidence intervals (CI). All statistical analyses were performed using SPSS (IBM® SPSS® Statistics Version 27). Following the recommendation of Bakker et al. [33], we compared effect sizes of the present study with those of similar studies instead of rigid benchmarks. For that purpose, Cohen’s *d* was calculated using G*Power (Version 3.1.0.6, Kiel University, Germany) and was interpreted according to Kinney et al. [34]: small: *d* = 0.14–0.30; medium: *d* = 0.31–0.55; large: *d* > 0.55.

## Results

**Fig. 1:**
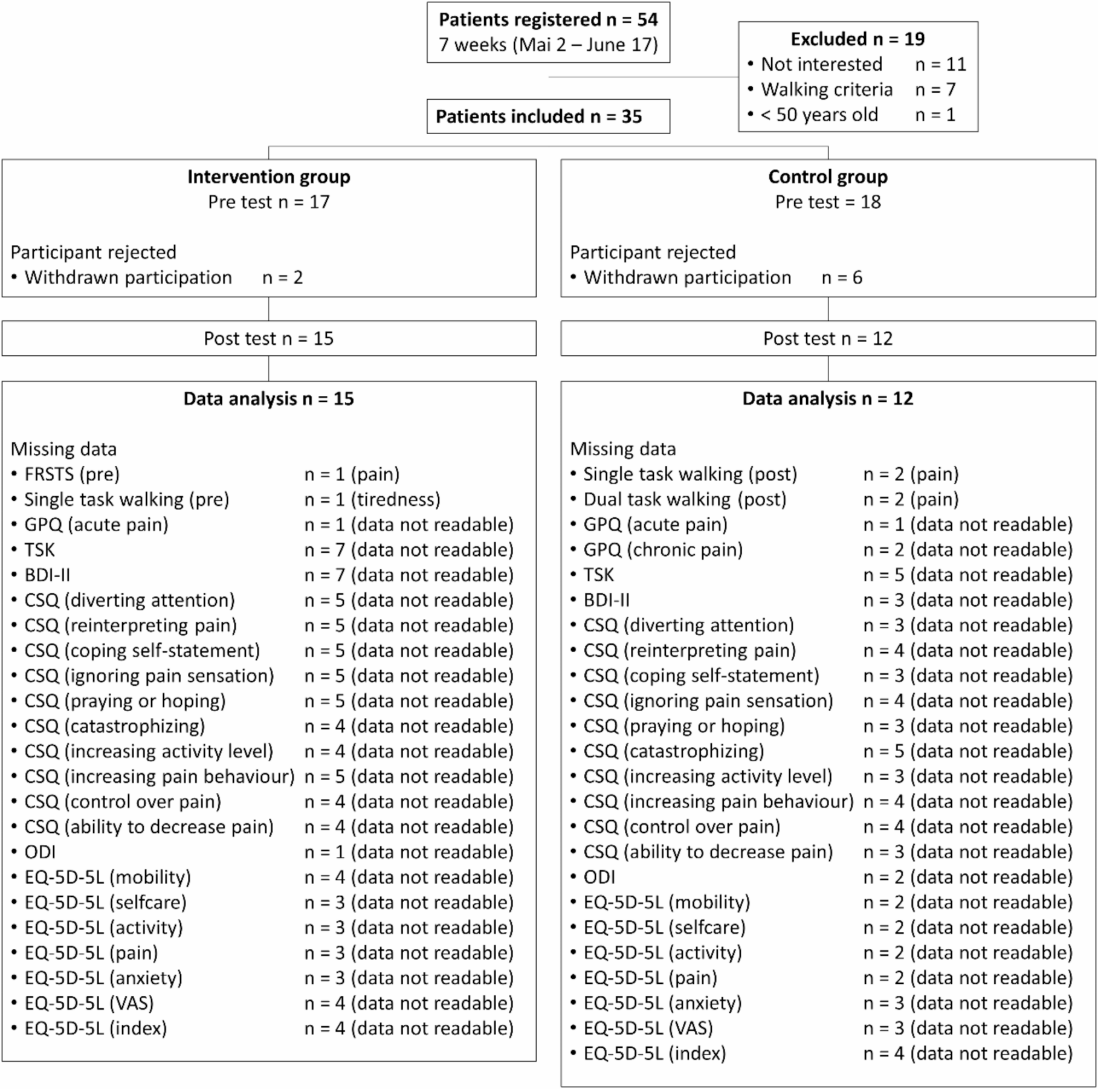
CONSORT flow diagram describing the recruitment process and the missing data for the statistical analysis FRSTS: Five-Repetition Sit-to-Stand Test; GPQ: German Pain Questionnaire; TSK: Tampa Scale of Kinesiophobia; BDI-II: Beck Depression Inventory-II; CSQ: Coping Strategies Questionnaire; ODI: Oswestry Disability Index; VAS: Visual analogue scale

In total, 54 patients registered and 35 individuals met the inclusion criteria and volunteered to participate in the study. Due to dropouts (refusal to perform the post-test), a final number of 27 patients suffering from specific CLBP (17 females, 10 males) were included in the statistical analysis. A detailed overview of all excluded patients and missing data is shown in the CONSORT flow diagram (Fig. 1).

The characteristics of the patients and group distribution are described in Table 2. Tables 3, 4 and 5, and 6 show the outcomes of the functional mobility, trunk ROM, leg extensor muscle power, and exercise capacity, single and dual task gait performance, executive performance as well as pain intensity, pain coping skills, psychosocial aspects, and quality of life, respectively, at pre-test.

**Table 2:** Patients’ characteristics. Data are shown as means (standard deviations)

	Intervention group	Control group	*p*
*n*	15 (10 females, 5 males)	12 (7 females, 5 males)	
Age [years]	63.8 (8.3)	61.6 (6.0)	0.116
Height [m]	1.69 (10)	1.67 (1)	0.126
Weight [kg]	81.7 (14.2)	83.0 (9.2)	0.314
Body Mass Index [kg/m²]	28.4 (4.5)	29.9 (3.0)	0.499

**Table 3:** Results for the functional tests performed at the pre-test. Data are shown as means (standard deviations [SD])

Variable		Pre-test			
		Intervention group		Control group	
		*n*	Mean (SD)	*n*	Mean (SD)
Timed Up and Go Test [s]		15	10.3 (2.4)	12	13.3 (3.1)
Trunk flexibility test					
	Sagittal ROM [°]	15	120.5 (18.0)	12	109.3 (25.1)
	Frontal ROM [°]	15	94.3 (22.2)	12	76.4 (24.1)
	Transversal ROM [°]	15	76.7 (23.3)	12	64.1 (25.2)
Five-Repetition Sit-to-Stand Test					
	Time [s]	14	14.5 (4.0)	12	16.0 (4.3)
	Relative leg extensor muscle power [W/kg]	14	2.7 (0.9)	12	2.0 (0.7)
Six-Minute Walk Test [m]		15	402.4 (102.0)	12	349.0 (112.6)

ROM: Range of motion

**Table 4:** Results for the single and dual task gait performance assessments at the pre-test. Data are shown as means (standard deviations [SD])

Variable		Pre-test			
		Intervention group		Control group	
		*n*	Mean (SD)	*n*	Mean (SD)
Single task					
	Stride length [m]	14	1.28 (0.15)	10	1.16 (0.10)
	Velocity [m/s]	14	1.18 (0.18)	10	1.04 (0.17)
	Minimum toe clearance [cm]	14	2.32 (0.51)	10	2.18 (0.45)
	Stride length CoV [%]	14	13.1 (1.9)	10	12.8 (2.9)
	Velocity CoV [%]	14	17.1 (2.3)	10	16.6 (2.2)
	Minimum toe clearance CoV [%]	14	29.9 (7.2)	10	31.8 (13.5)
	Arithmetic task performance	14	52.2 (15.7)	12	34.5 (14.6)
Dual task					
	Stride length [m]	14	1.24 (0.16)	10	1.07 (0.09)
	Velocity [m/s]	14	1.05 (0.22)	10	0.86 (0.16)
	Minimum toe clearance [cm]	14	2.17 (0.53)	10	1.88 (0.28)
	Stride length Cov [%]	14	13.0 (1.9)	10	13.2 (1.5)
	Velocity CoV [%]	14	17.6 (2.1)	10	18.3 (2.2)
	Minimum toe clearance CoV [%]	14	30.0 (8.5)	10	39.3 (10.6)
	Arithmetic task performance	14	45.5 (14.2)	10	30.0 (13.5)
Dual task costs					
	Stride length [%]	14	3.9 (5.2)	10	8.1 (5.5)
	Velocity [%]	14	13.0 (13.4)	10	16.4 (11.3)
	Minimum toe clearance [%]	14	7.0 (9.9)	10	12.4 (9.4)
	Stride length CoV [%]	14	-1.1 (20.6)	10	6.9 (20.9)
	Velocity CoV [%]	14	3.0 (11.3)	10	8.5 (14.5)
	Minimum toe clearance CoV [%]	14	-0.5 (11.9)	10	17.2 (26.6)
	Arithmetic task performance [%]	14	12.8 (17.3)	10	10.8 (29.3)

CoV: Coefficient of variation

**Table 5:** Results for the executive function tests performed at the pre-test. Data are shown as means (standard deviations [SD])

Variable		Pre-test			
		Intervention group		Control group	
		*n*	Mean (SD)	*n*	Mean (SD)
Stroop Color and Word Test					
	Interference score	15	-21.8 (14.5)	12	-13.9 (21.5)
Trail Making Test					
	Part A [s]	15	34.1 (9.6)	12	42.3 (15.6)
	Part B [s]	15	96.6 (59.5)	12	116.8 (67.5)
	Part B– Part A [s]	15	64.5 (64.4)	12	74.6 (68.1)

***INSERT Table 6***

Physical functions

**Table 6:** Results of the questionnaires at the pre-test. Data are shown as means (standard deviations [SD])

Variable		Pre-test			
		Intervention group		Control group	
		*n*	Mean (SD)	*n*	Mean (SD)
Pain intensity					
	Acute (GPQ)	14	4.3 (2.7)	11	6.1 (2.2)
	Chronic (GPQ)	15	5.9 (2.1)	10	6.2 (2.1)
	During the trunk flexibility test	15	2.8 (2.1)	12	3.8 (2.1)
Kinesiophobia (TSK)		10	20.2 (6.2)	7	20.3 (5.2)
Depression (BDI-II)		10	12.7 (6.9)	9	11.4 (8.6)
Cognitive coping strategies (CSQ)					
	Diverting attention	12	1.5 (1.3)	9	2.2 (1.2)
	Reinterpreting pain	12	0.7 (0.9)	8	0.5 (0.5)
	Coping self-statement	12	2.9 (1.3)	9	3.2 (1.0)
	Ignoring pain sensation	12	1.9 (1.3)	8	1.6 (0.9)
	Praying or hoping	12	1.7 (0.8)	9	2.5 (0.5)
	Catastrophizing	13	2.2 (1.1)	7	2.0 (1.4)
Behavioral coping strategies (CSQ)					
	Increasing activity level	13	2.2 (1.1)	9	2.5 (0.7)
	Increasing pain behavior	12	2.6 (0.7)	8	3.2 (0.7)
Effectiveness rating (CSQ)					
	Control over pain	13	1.4 (2.2)	8	0.4 (0.5)
	Ability to decrease pain	13	2.4 (2.2)	9	1.4 (1.5)
Disability (ODI)		14	27.1 (11.4)	10	34.3 (10.5)
Quality of life (EQ-5D-5 L)					
	Mobility	13	2.0 (0.9)	10	2.6 (1.1)
	Selfcare	14	1.1 (0.4)	10	1.7 (0.8)
	Activity	14	2.0 (0.8)	10	2.4 (1.0)
	Pain	14	2.7 (0.8)	10	3.2 (0.8)
	Anxiety	14	1.7 (0.9)	9	1.7 (0.9)
	VAS	13	61.2 (18.6)	9	50.3 (17.9)
	Index	13	0.8 (0.2)	8	0.6 (0.3)

GPQ: German Pain Questionnaire; TSK: Tampa Scale of Kinesiophobia; BDI-II: Beck Depression Inventory-II, CSQ: Coping Strategies Questionnaire; ODI: Oswestry Disability Index; VAS: Visual analogue scale

### Physical functions

**Fig. 2:**
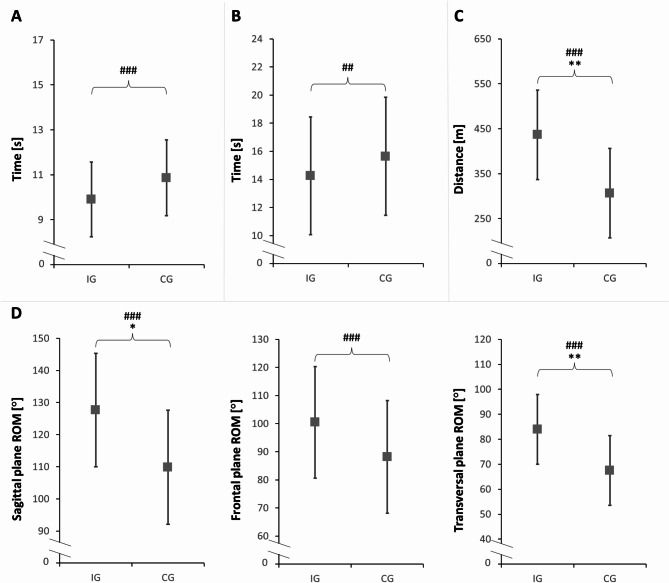
Means and standard deviations for (**A**) Timed Up and Go Test, (**B**) Five-Repetition Sit-to-Stand Test, (**C**) Six-Minute Walk Test, and (**D**) Trunk Flexibility Test of both groups at post-test ROM: Range of motion; IG: Intervention group; CG: Control group. *p ≤ 0.05; **p ≤ 0.01; ^##^*d* = 0.31–0.55; ^###^: *d* > 0.55

The ANCOVA with baseline–adjustment indicated a significant increase of trunk ROM (sagittal and transversal planes) in the IG compared to CG with large effect sizes. Additionally, exercise capacity was also significantly higher in the IG compared to CG with a large effect size. No significant differences were found for trunk ROM in the frontal plane, TUG Test performance, and leg extensor muscle power between the IG and CG (Fig. 2; Table 7).

**Table 7:** Adjusted means (adjusted standard deviations) and outcomes of the ANCOVA with baseline-adjustment for the functional mobility, trunk ROM, leg extensor muscle power, and exercise capacity tests performed at the post-test

Variable		Post-test		IG-CG	95% CI	ANCOVA			d
		IG	CG			F	df	*p*	
Timed Up and Go Test [s]		9.9 (1.7)	10.9 (1.7)	-1.0	[-2.4, 0.5]	1.9	1, 24	0.176	**0.59**
Trunk flexibility test									
	Sagittal ROM [°]	127.7 (17.7)	109.8 (17.8)	17.8	[3.4, 32.2]	6.5	1, 24	**0.018**	**1.00**
	Frontal ROM [°]	100.4 (19.9)	88.2 (20.0)	12.3	[-4.3, 28.8]	2.3	1, 24	0.139	**0.61**
	Transversal ROM [°]	83.9 (13.9)	67.5 (14.0)	16.4	[5.0, 27.7]	8.9	1, 24	**0.006**	**1.18**
Five-Repetition Sit-to-Stand Test									
	Time [s]	14.3 (4.2)	15.6 (4.2)	-1.4	[-4.9, 2.2]	0.7	1, 23	0.428	0.31
	Relative leg extensor muscle power [W/kg]	2.8 (0.7)	2.3 (0.7)	0.4	[-0.2, 1.0]	2.2	1, 23	0.148	**0.71**
Six-Minute Walk Test [m]		436.9 (99.5)	306.9 (99.8)	130.0	[49.1, 210.9]	11.0	1, 24	**0.003**	**1.30**

ANCOVA: Analysis of covariance; IG: Intervention group; CG: Control group; IG - CG: Mean difference between the intervention and control group; CI: Confidence interval; ROM: Range of motion; **bold**: *p* ≤ 0.05 and/or *d* > 0.55

### Single and dual task gait performance

The ANCOVA with baseline-adjustment revealed no significant differences for the spatio-temporal gait parameters at the post-test during both the single and dual task walking. Similar results were found for the cognitive task during both conditions. However, the DTC for the minimum toe clearance CoV were significantly lower in the IG compared to the CG with a large effect size (Table 8).

**Table 8:** Adjusted means (adjusted standard deviations) and outcomes of the ANCOVA with baseline-adjustment for the single and dual task performance assessments at the post-test

Variable		POST		IG - CG	95% CI	ANCOVA			d
		IG	CG			F	df	*p*	
Single task									
	Stride length [m]	1.30 (0.10)	1.27 (0.10)	0.03	[-0.06, 0.12]	0.6	1, 22	0.456	0.30
	Velocity [m/s]	1.23 (0.13)	1.26 (0.13)	0.08	[-0.04, 0.19]	1.8	1, 22	0.196	0.23
	Minimum toe clearance [cm]	2.35 (0.35)	2.27 (0.35)	0.08	[-0.21, 0.38]	0.3	1, 22	0.568	0.23
	Stride length CoV [%]	12.9 (1.6)	11.7 (1.6)	1.2	[-0.1, 2.5]	3.4	1, 22	0.078	**0.75**
	Velocity CoV [%]	16.4 (1.5)	16.0 (1.5)	0.4	[-0.9, 1.6]	0.4	1, 22	0.547	0.27
	Minimum toe clearance CoV [%]	33.1 (8.5)	27.1 (8.5)	6.0	[-1.1, 13.2]	3.0	1, 22	0.095	**0.71**
	Arithmetic task performance	47.7 (8.8)	51.4 (8.9)	-3.7	[-11.3, 3.9]	1.0	1, 24	0.324	0.42
Dual task									
	Stride length [m]	1.19 (0.08)	1.20 (0.08)	-0.01	[-0.08, 0.07]	0.0	1, 21	0.837	0.13
	Velocity [m/s]	1.03 (0.11)	1.03 (0.11)	0.0	[-0.10, 0.10]	0.0	1, 21	0.997	0.00
	Minimum toe clearance [cm]	1.98 (0.30)	2.14 (0.30)	-0.16	[-0.42, 0.11]	1.5	1, 21	0.228	0.53
	Stride length CoV [%]	12.9 (2.0)	12.5 (2.0)	0.4	[-1.3, 2.1]	0.2	1, 21	0.654	0.20
	Velocity CoV [%]	16.9 (1.8)	16.5 (1.8)	0.4	[-1.1, 1.9]	0.3	1, 21	0.573	0.22
	Minimum toe clearance CoV [%]	32.8 (8.2)	29.5 (8.3)	3.3	[-4.2, 10.8]	0.9	1, 21	0.365	0.40
	Arithmetic task performance	45.3 (7.1)	38.8 (7.3)	6.6	[-0.0, 13.2]	4.3	1, 21	0.051	**0.90**
Dual task costs									
	Stride length [%]	7.7 (4.5)	6.1 (4.6)	1.6	[-2.4, 5.6]	0.7	1, 22	0.409	0.35
	Velocity [%]	15.5 (6.6)	13.3 (6.6)	2.2	[-3.4, 7.8]	0.6	1, 22	0.434	0.33
	Minimum toe clearance [%]	13.5 (7.0)	7.6 (7.1)	6.0	[-0.1, 12.1]	4.1	1, 22	0.055	**0.84**
	Stride length CoV [%]	-2.5 (19.1)	6.4 (19.2)	-8.9	[-25.3, 7.4]	1.3	1, 22	0.271	0.46
	Velocity CoV [%]	-1.4 (12.5)	4.9 (12.5)	-6.3	[-17.0, 4.4]	1.5	1, 22	0.236	0.50
	Minimum toe clearance CoV [%]	-11.6 (26.5)	14.3 (27.0)	-25.9	[-49.7, -2.2]	5.1	1, 22	**0.034**	**0.97**
	Arithmetic task performance [%]	7.8 (16.3)	20.4 (16.3)	-12.6	[-26.6, 1.4]	3.5	1, 21	0.076	**0.77**

ANCOVA: Analysis of covariance; IG: Intervention group; CG: Control group; IG - CG: Mean difference between the intervention and control group; CI: Confidence interval; CoV: Coefficient of variation; **bold**: *p* ≤ 0.05 and/or *d* > 0.55

### Executive functioning

No significant differences between groups at the post-test were found for the interference score and the Trail Making Test performance (Table 9).

**Table 9:** Adjusted means (adjusted standard deviations) and outcomes of the ANCOVA with baseline-adjustment for the executive function tests at the post-test

Variable		POST		IG - CG	95% CI	ANCOVA				d
		IG	CG			F	df	*p*		
Stroop Color and Word Test										
	Interference score	-11.3 (12.1)	-9.6 (12.2)	-1.7	[-11.5, 8.1]	0.1	1, 24	0.725		0.14
Trail Making Test										
	Part A [s]	31.1 (9.4)	36.6 (9.4)	-5.5	[-13.2, 2.2]	2.2	1, 24	0.152		**0.59**
	Part B [s]	89.8 (19.2)	87.6 (19.2)	2.3	[-13.2, 17.7]	0.1	1, 24	0.763		0.11
	Part B– Part A [s]	56.5 (17.7)	53.7 (17.7)	2.8	[-11.4, 17.0]	0.2	1, 24	0.687		0.16

ANCOVA: Analysis of covariance; IG: Intervention group; CG: Control group; IG - CG: Mean difference between the intervention and control group; CI: Confidence interval; **bold**: *d* > 0.55

### Pain intensity, pain coping skills, psychosocial aspects, disability, and quality of life

The ANCOVA with baseline-adjustment demonstrated that chronic pain was significantly lower in the IG compared to the CG at the post-test with a large effect size. Additionally, kinesiophobia and depression were both significantly reduced in the IG compared to the CG with large effect sizes. The pain coping strategies, disability, and quality of life did not significantly differ between both groups at post-test (Table 10).

**Table 10:** Adjusted means (adjusted standard deviations) and outcomes of the ANCOVA with baseline-adjustment for the questionnaires filled in at the post-test

Variable		POST		IG - CG	95% CI	ANCOVA				d
		IG	CG			F	df	*p*		
Pain intensity										
	Acute (GPQ)	3.0 (2.1)	4.1 (2.2)	-1.1	[-2.9, 0.8]	1.5	1, 22	0.235		0.51
	Chronic (GPQ)	3.6 (1.8)	6.0 (1.8)	-2.4	[-4.0, -0.8]	10.2	1, 22	**0.004**		**1.33**
	During the trunk flexibility test	2.4 (1.8)	3.5 (1.8)	-1.2	[-2.7, 0.3]	2.7	1, 24	0.113		**0.61**
Kinesiophobia (TSK)		18.4 (3.3)	22.2 (3.3)	-3.8	[-7.3, -0.3]	5.4	1, 14	**0.035**		**1.15**
Depression (BDI-II)		4.7 (4.8)	9.9 (4.8)	-5.2	[-9.8, -0.5]	5.4	1, 16	**0.034**		**1.08**
Cognitive coping strategies (CSQ)										
	Diverting attention	2.1 (0.7)	1.7 (0.7)	0.4	[-0.3, 1.1]	1.5	1, 18	0.239		**0.57**
	Reinterpreting pain	0.8 (0.5)	0.7 (0.5)	0.1	[-0.4, 0.5]	0.1	1, 17	0.753		0.20
	Coping self-statement	3.1 (0.7)	2.9 (0.7)	0.1	[-0.5, 0.8]	0.2	1, 18	0.654		0.29
	Ignoring pain sensation	2.1 (0.8)	1.8 (0.8)	0.4	[-0.4, 1.1]	1.2	1, 17	0.286		0.38
	Praying or hoping	1.5 (0.7)	2.0 (0.7)	-0.5	[-1.2, 0.2]	2.0	1, 18	0.174		**0.71**
	Catastrophizing	1.5 (0.7)	1.8 (0.7)	-0.3	[-1.0, 0.4]	0.7	1, 17	0.418		0.43
Behavioral coping strategies (CSQ)										
	Increasing activity level	2.5 (0.7)	2.2 (0.7)	0.3	[-0.4, 0.9]	0.8	1, 19	0.378		0.43
	Increasing pain behavior	3.0 (0.6)	2.8 (0.6)	0.2	[-0.4, 0.8]	0.5	1, 17	0.476		0.33
Effectiveness rating (CSQ)										
	Control over pain	1.0 (0.8)	1.0 (0.8)	-0.0	[-0.8, 0.7]	0.1	1, 18	0.894		0.00
	Ability to decrease pain	1.9 (1.6)	1.5 (1.6)	0.3	[-1.2, 1.8]	0.2	1, 19	0.667		0.25
Disability (ODI)		24.5 (6.2)	27.0 (6.2)	-2.5	[-8.0, 3.0]	0.9	1, 21	0.362		0.40
Quality of life (EQ-5D-5 L)										
	Mobility	1.9 (0.6)	2.4 (0.6)	-0.5	[-1.0, 0.1]	3.1	1, 20	0.093		**0.83**
	Selfcare	1.4 (0.7)	1.8 (0.7)	-0.4	[-1.1, 0.3]	1.4	1, 21	0.243		**0.57**
	Activity	1.8 (0.8)	2.0 (0.8)	-0.3	[-1.0, 0.4]	0.7	1, 21	0.409		0.25
	Pain	2.4 (0.7)	2.4 (0.7)	-0.0	[-0.6, 0.5]	0.0	1, 21	0.868		0.00
	Anxiety	1.1 (0.6)	1.6 (0.6)	-0.4	[-0.9, 0.1]	3.2	1, 20	0.090		**0.83**
	VAS	63.7 (13.6)	61.5 (13.8)	2.2	[-10.6, 14.9]	0.1	1, 19	0.727		0.16
	Index	0.9 (0.1)	0.8 (0.1)	0.1	[-0.0, 0.2]	2.6	1, 18	0.127		**1.00**

ANCOVA: Analysis of covariance; IG: Intervention group; CG: Control group; CG - IG: Mean difference between control and intervention; CI: Confidence interval; GPQ: German Pain Questionnaire; TSK: Tampa Scale of Kinesiophobia; BDI-II: Beck Depression Inventory-II, CSQ: Coping Strategies Questionnaire; ODI: Oswestry Disability Index; VAS: Visual analogue scale; **bold**: *p* ≤ 0.05 and/or *d* > 0.55

## Discussion

The primary objective of this pilot study was to investigate if the MultiMove intervention in addition to a standard inpatient therapy is more effective to improve functional mobility compared to the standard inpatient therapy alone in patients with specific CLBP. In contrast to our hypotheses, no significant improvement in TUG performance in favor of the patients who carried out the MultiMove intervention was found. Nevertheless, significant between-group differences were found for the secondary outcomes trunk ROM (in particular in the sagittal and transversal plane) as well as exercise capacity. Although the spatio-temporal gait parameters did not significantly differ between groups, lower DTC were observed for the minimum toe clearance CoV. Moreover, chronic pain intensity, kinesiophobia, and depression were significantly reduced in the IG compared to the CG.

To our knowledge, MultiMove is the first multimodal intervention that has combined motor-cognitive and dance exercises, performed successively during each training sessions, to improve clinical and functional outcomes after a 3-week inpatient rehabilitation in patients with specific CLBP. Consequently, the comparison with existing training modalities for CLBP is somewhat challenging. The following discussion will therefore compare the effects of the MultiMove intervention on the respective outcomes with the results of studies that have investigated the effects of dance, motor skill, and motor-cognitive training on the different outcomes.

### Physical functions

Although, TUG performance (primary outcome) was not improved in the group with an additional MultiMove intervention compared to the standard care alone, trunk ROM as well as 6MWT performance were significantly increased with large effect sizes. These results are in accordance with the outcome of a recent meta-analysis highlighting the beneficial effects of dance interventions on physical functions in healthy older adults [35]. Indeed, it has been demonstrated that dance training can effectively increase flexibility, muscular strength, endurance, and balance performance, which are required for the completion of daily tasks. Moreover, a 6-week motor skill-based training (i.e., person-specific exercises to modify the altered movement pattern during functional activities) has also led to a higher performance during a'picking up an object' task compared to strength and flexibility training in CLBP patients [36].

### Single and dual task gait performance

Data of the present study indicate that the spatio-temporal gait parameters, such as stride length and gait velocity, were not altered by the MultiMove intervention. This finding was unexpected given that a meta-analysis by Fong Yan et al. [37], which compared the effect of dance interventions with non-dance interventions on several health-related outcomes, showed an increase in gait velocity, especially in elderly, obese, and/or type 2 diabetes populations. Nevertheless, the DTC of the minimum toe clearance CoV were lower in the IG compared to the CG suggesting a reduced minimum toe clearance variability during walking while simultaneously performing a cognitive task compared to walking only. This might be attributable to the motor-cognitive and/or dance training that requires the simultaneous execution of motor and cognitive tasks [13–15]. Given that the variability of the minimum toe clearance was negatively associated with brain activity in the dorsolateral prefrontal cortex in CLBP patients [38], which is involved in cognitive, affective, and sensory processing [39], it might be that neuroplastic changes induced by the additional MultiMove intervention contributed to the reduced DTC.

### Executive functioning

Two core executive functions, that are the inhibitory control and the cognitive flexibility [40], were assessed in this study. In contrast to our hypotheses, data analysis did not reveal significant differences between groups in cognitive task performance. Given that motor-cognitive and dance training performed over several months have been shown to increase cognitive performance [15, 41, 42], this finding might be related to the duration of the MultiMove intervention, which lasted only 3 weeks. Consequently, it might be that a longer MultiMove intervention and/or a higher training volume could have improved executive functioning.

### Pain intensity

Chronic pain intensity (i.e., perceived pain intensity over the last weeks) was significantly reduced with a large effect size in the IG compared to the CG. This finding is in line with the outcome of a recent review, which deemed dancing as an effective adjunct to reduce chronic pain across diverse populations [43]. Pain, especially chronic pain, is mainly related to musculoskeletal conditions but also to other psychosocial aspects such as stress, deconditioning, fear, catastrophizing, as well as feelings of isolation and separation. In this regard, Hickman et al. [43] suggested that the mechanisms leading to the pain reduction include the physical (e.g., improved musculoskeletal and cardiovascular function) and psychosocial benefits of dancing (e.g., socialization, in-group bonding, touch). Especially the latter might improve mood, self-confidence, and pain thresholds. Additionally, it has been shown that chronic pain is associated with functional and structural impairments in several brain areas [39], which might be positively influenced by the neuroplasticity promoted by motor-cognitive and/or dancing exercises [15, 42], thereby potentially reducing pain intensity.

### Pain coping skills, psychosocial aspects, disability, and quality of life

Although the pain coping strategies, disability, and quality of life did not differ between groups after the interventions, lower kinesiophobia and depression were found in favor of the IG. Regarding kinesiophobia, the motor-cognitive and/or dancing exercises performed during the MultiMove intervention could have contributed to this finding, given that dance/movement therapies have been suggested to increase self-awareness in patients with chronic pain. This includes realizing that they are able to move better and that pain is not as bad as they thought as well as that movement can reduce pain, which collectively might promote cognitive and emotional restructuring with regard to physical activity and pain [44]. Depression was also reduced after the MultiMove intervention compared to the standard care. This is in line with the findings of a recently published umbrella review indicating that physical activity interventions are effective to reduce depression, anxiety, and distress in healthy adults as well as people with mental health disorders and chronic diseases. It is thought that the beneficial effects of exercise interventions on depression and anxiety are related to various psychological, neurophysiological, and social mechanisms [45]. Furthermore, especially dancing is assumed to reduce depression due to the potential pleasure generated by the dance movements and the social interaction [46]. Moreover, dancing could be more attractive and inclusive than other exercise interventions, which might enhance the adherence and compliance of patients to the intervention [37].

Based on the effect sizes, the additional MultiMove intervention had further positive effects regarding several other outcomes that were, however, not significantly different to the standard care. Therefore, conducting a full-scaled randomized controlled clinical trial with an adequate sample size is recommended to verify and extent the results of the present study. Moreover, previous randomized controlled trials primarily investigated the effects of multimodal interventions on pain intensity or functional mobility as primary outcomes [47–50]. However, the role of cognitive functioning (including executive functions) in chronic pain is sometimes overlooked. Therefore, we recommend including cognitive/executive function tests and/or brain imaging techniques in future studies to unravel the relationship between cognitive/executive functions and CLBP.

### Limitations

Given that MultiMove was designed as a group therapy, patients were assigned to the IG and CG based on their admission day and not via randomization. A second limitation was that the MultiMove intervention was an addition to the standard rehabilitation program and not a substitute. Consequently, the differences between groups in the clinical and functional outcomes might be related to a higher training volume. However, the recommended training volume for CLBP rehabilitation is 2–3 sessions per week, 60–90 min each [51], resulting in 360–810 min of training, when calculated for a duration of 3 weeks. Additionally, the average training volume performed by the CG during the 3-week standard inpatient rehabilitation was 1755 min (mean training volume per week = 585 min). In the present study, the patients took part in 9–12 MultiMove sessions, which resulted in 270–360 min of training. Thus, the improvements highlighted above are more likely to be related to the exercise type rather than to a higher training volume. Third, although the interventions performed during standard care followed a standardized program, deficit-oriented individual adjustments were made (e.g., intensity and duration), which could have influenced the outcome of the present study.

## Conclusion

The present data did not reveal any negative effects related to the addition of MultiMove to a standard inpatient rehabilitation program. Therefore, MultiMove is a safe new multimodal intervention with short, easy-to-implement exercises with adaptative difficulty, which led to improvements in trunk ROM, exercise capacity, DTC during walking, chronic pain intensity, kinesiophobia, and depression in patients with specific CLBP.

## Data Availability

Raw data are available from the corresponding author on request.

## Abbreviations

6MWT: Six-Minute Walk Test
ANCOVA: Analysis of covariance
CG: Control group
CI: Confidence interval
CLBP: Chronic low back pain
CoV: Coefficient of variation
DTC: Dual task cost
FRSTS: Five-Repetition Sit-to-Stand
IG: Intervention group
IMU: Inertial measurement units
LBP: Low back pain
ROM: Range of motion
SD: Standard deviation
TUG: Timed Up and Go
